# The advantage of one‐step nucleic acid amplification for the diagnosis of lymph node metastasis in colorectal cancer patients

**DOI:** 10.1002/ags3.12392

**Published:** 2020-08-29

**Authors:** Yukiharu Hiyoshi, Takashi Akiyoshi, Yosuke Fukunaga

**Affiliations:** ^1^ Gastroenterological Center Department of Gastroenterological Surgery The Cancer Institute Hospital of Japanese Foundation for Cancer Research Tokyo Japan

**Keywords:** CK‐19, colorectal cancer, lymph node metastasis, micrometastasis, OSNA, sentinel lymph node

## Abstract

Generally, the postoperative examination of lymph nodes (LNs) is based on a microscopic examination of one hematoxylin and eosin (HE)‐stained slide; however, an examination of only one part of the LN might lead to incorrect staging of the tumor due to tissue allocation bias. Although multilevel sectioning and the use of immunohistochemistry (IHC) have improved the detection of micrometastases in LNs, this approach is laborious, time‐consuming, and costly. A novel molecular technique for the detection of LN metastases of tumors, called one‐step nucleic acid amplification (OSNA), is a rapid and semi‐quantitative examination quantifying the number of cytokeratin 19 (CK‐19) mRNA copies derived from a tumor. OSNA is already in clinical use for the diagnosis of LN metastasis in breast cancer patients; however, the use of OSNA is under investigation with promising results for colorectal cancer (CRC). The present review assessed recent studies on OSNA vs a histopathological examination and its implications for CRC staging and treatment. A total of 16 studies of OSNA in CRC yielded by a PubMed search were reviewed. Among them, seven studies evaluating the diagnostic performance revealed that OSNA had a high specificity (96.8%), high concordance rate (96.0%), and negative predictive value (98.6%) in a pooled assessment. In addition, four studies examining the utility of OSNA in sentinel LNs (SLNs) and two studies focusing on upstaging in pathologically node‐negative CRC patients were also reviewed. Multicenter prospective studies with a large cohort of CRC patients are warranted to reveal the benefits of OSNA in the future.

## Graphical abstract

Although the validity of one‐step nucleic acid amplification (OSNA) assay for detecting lymph node metastasis has been widely reported in patients with other types of malignancies, such as colorectal cancer (CRC), lung cancer, gastric cancer, head and neck squamous cell carcinoma, and thyroid cancer, its clinical benefit has not been established. We herein review studies relevant to OSNA in CRC and discuss perspectives on the future applications of this assay.

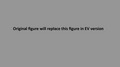

## 1 INTRODUCTION

Colorectal cancer (CRC) is the third most common cancer worldwide.^1^ The lymph node (LN) is the most common site of metastasis, and the LN status is the most powerful prognostic factor.^2^ The Guidelines for Tumor Node Metastasis (TNM) staging are provided by the American Joint Committee on Cancer (AJCC) and the Union of International Cancer Control (UICC). LN (N category) staging includes information on whether or not the cancer has spread to regional LNs and how many LNs are involved. Isolated tumor cells (ITCs) in LNs are defined as single cancer cells or small clusters of tumor cells measuring ≤0.2 mm and classified as N0 (i+). Micrometastases are defined as tumor clusters measuring >0.2 mm but ≤2.0 mm in their greatest dimension and classified as N1 (mic). Although the prognostic value of ITCs is unclear, a recent systematic review and meta‐analysis showed micrometastasis to be a significantly poor prognostic factor.^3^ Per the AJCC 8th edition, the micrometastasis may be designated as N1 (mic), but that may be better considered standard positive LNs. Because of the strong prognostic relevance of LN metastases in CRC, the presence of occult tumor cells, defined as micrometastases or ITCs within regional LNs that are not detected on a conventional histopathologic examination using hematoxylin and eosin (HE) staining has been suspected to be a marker of systemic tumor spread in these patients.^4^ Therefore, the detection of occult disease may help identify patients with node‐negative CRC who are at a high risk of tumor recurrence and who might benefit from adjuvant therapy.

Combining routine HE staining with tumor‐specific immunohistochemistry (IHC) using step sectioning specimens or molecular detection techniques, such as reverse transcriptase polymerase chain reaction (RT‐PCR), provides a higher sensitivity than routine histology alone.^5^, ^6^ However, these methods are burdensome and time‐consuming, so a quick, highly sensitive, and specific diagnostic technique that enables a prompt intraoperative examination is necessary.

A new molecular technique called one‐step nucleic acid amplification (OSNA), which is a rapid and semi‐quantitative intraoperative procedure for quantifying the number of cytokeratin 19 (CK‐19) mRNA copies in LNs, has been employed to assess the LN progression of tumors. OSNA was first reported in breast cancer patients and has been shown to be effective for detecting nodal metastases.^7^ OSNA can assess the occurrence of metastasis in the whole LN regardless of tissue allocation biases and estimate the total volume of tumor cells semi‐quantitatively. This is one important advantage of OSNA over a pathological assessment.

Although the validity of OSNA assay for detecting LN metastasis has also been widely reported in patients with other types of malignancies, such as CRC, lung cancer, gastric cancer, head and neck squamous cell carcinoma (HNSCC), and thyroid cancer, its clinical benefit has not been established.^8^, ^9^ In addition, several studies have recently evaluated OSNA for LN metastasis in CRC, focusing on pathological stage II disease, sentinel LN (SLN) metastasis, and lateral pelvic LN (LPLN) metastasis.

We herein review studies relevant to OSNA in CRC and discuss perspectives on the future applications of this assay.

## 2 LITERATURE SEARCH

A search for medical reports relevant to OSNA in colorectal cancer published before the end of June 2020 was made through PubMed using the keywords of “OSNA” and “colorectal cancer” or “colon cancer” or “rectal cancer.” Reports written in languages other than English were excluded. Review articles were also excluded. The 16 studies obtained by the PubMed search are shown in Table 1.

**Table 1:** Characteristics of OSNA studies in CRC patients

Author	Year	Patients number(Sample number)	Tumor type	Purpose of the OSNA analysis
Croner et al^10^	2010	184 (184)	Colorectal	Diagnosis of LN metastasis
Yamamoto et al^11^	2011	85 (385)	Colorectal	Diagnosis of LN metastasis
Guller et al^12^	2012	22 (313)	Colon	Diagnosis of LN metastasis
Yamamoto et al^13^	2013	30 (66)	Colorectal	Identification of CK19
Vogelaar et al^14^	2014	128 (325)	Colon	Diagnosis of SLN metastasis
Yamamoto et al^15^	2016	204 (1925)	Colorectal	Diagnosis of LN metastasis
Rakislova et al^16^	2017	188 (3206)	Colon	Diagnosis of pooled LN metastasis
Miyake et al^17^	2017	25 (306)	Rectum	Indication of LPLN dissection
Marhic et al^18^	2017	17	Colon	Diagnosis of SLN metastasis
Colling et al^19^	2017	19 (82)	Colorectal	Diagnosis of LN metastasis
Aldecoa et al^20^	2017	71 (936)	Colon	OSNA with endoscopic tattooing
Yeung et al^21^	2018	16 (78)	Colorectal	OSNA with ICG detection
Brito et al^22^	2018	59 (753)	Colon	Pathologically node‐negative CRC
Esposito et al^23^	2019	34 (51)	Colorectal	Diagnosis of SLN metastasis
Diaz‐Mercedes et al^24^	2019	17 980	Colorectal	Budget impact analysis
Itabashi et al^25^	2020	195	Colorectal	Pathologically node negative CRC

Abbreviations: CRC, colorectal cancer; ICG, indocyanine green; LPLN, lateral pelvic lymph node; OSNA, one‐step nucleic acid amplification; SLN, sentinel lymph node.

## 3 THE IDENTIFICATION OF AN OPTIMAL mRNA MARKER FOR THE OSNA ASSAY IN CRC

At present, the validity of the OSNA assay targeting CK19 mRNA for detecting LN metastasis has been widely reported in patients with various types of tumors, including CRC. Yamamoto et al reported the background for the identification of CK19 mRNA as an optimal marker for the OSNA assay in CRC.^13^ Ninety‐eight candidate mRNAs selected from the genome‐wide expressed sequence tag database were evaluated by quantitative RT‐PCR using a mixture of metastasis‐positive LNs and another mixture of metastasis‐negative LNs in CRC patients. Thereafter, the three identified candidates (CK19, CEA, and CK20) were examined by an OSNA assay, and CK19 mRNA was found to have the best diagnostic performance. The cut‐off value for discriminating positive from negative LNs was set at 75‐500 copies/µL, with 96.4% sensitivity and 100% specificity.

## 4 DIAGNOSTIC PERFORMANCE OF THE OSNA ASSAY IN CRC

Among the 16 studies listed in Table 1, the seven comparing the diagnostic performance between the OSNA and a pathological examination for the detection of LN metastasis in CRC are shown in Table 2. The calculated values of sensitivity, specificity, concordance, positive predictive value (PPV), and negative predictive value (NPV) in a pooled analysis were 90.4%, 96.8%, 96.0%, 79.8%, and 98.6%, respectively. These values are similar to those of breast cancer previously reviewed by Tamaki et al.^6^

**Table 2:** A comparison of the diagnostic accuracy of the OSNA assay in CRC patients

Author	Pathological evaluation	IHC	LN number inspected by OSNA (mean)	Sensitivity (%)	Specificity (%)	Concordance (%)	PPV (%)	NPV (%)
Croner et al^10^	Multi‐slice	CK19	1.0	92.5	96.5	93.6	88.1	97.9
Yamamoto et al^11^	Multi‐slice	None	4.5	95.2	97.7	97.1	91.9	98.7
Guller et al^12^	Multi‐slice	CK19	14.2	94.5	97.6	97.1	89.7	98.8
Yamamoto et al^15^	Single‐slice	None	9.4	86.2	96.5	95.7	66.5	98.8
Colling et al^19^	Single‐slice	None	4.3	92.9	97.1	96.3	86.7	98.5
Yeung et al^21^	Single‐slice	None	4.9	100	98.4	98.7	94.1	100
Esposito et al^23^	Multi‐slice	None	1.5	69.2	100	88.2	100	84.0
Total	—	—	5.4	90.4	96.8	96.0	79.8	98.6

Abbreviations: CK19, cytokeratin 19; IHC, immunohistochemistry; NPV, negative predictive value; PPV, positive predictive value.

It should be noted that the methodology of the histopathological evaluation for detection of the LN metastasis differed among studies. For example, a standard pathological examination using HE staining with a single 4‐µm‐thick tissue section was used in some studies,^15^, ^19^, ^21^ while a more precise pathological evaluation for the detection of LN metastasis, such as macrometastasis, micrometastasis, and isolated tumor cells (ITCs), was attempted, and the diagnostic accuracy was compared between an intensive pathological evaluation and the OSNA assay in others.^10^, ^11^, ^12^, ^23^ Studies conducted by Yamamoto et al^11^ and Yeung et al^21^ pathologically evaluated LN metastasis using HE staining with multi‐sliced tissue sections, and Croner et al^26^ examined LNs using not only HE staining but also CK19 IHC. Guller et al compared the performance of the OSNA with both standard HE staining (single section) and intensive histopathology (HE staining and CK19 IHC with 0.2‐mm skip‐sliced sections) in the detection of colon cancer LN metastasis, and the OSNA had a similar performance for detecting LN metastasis to intensive histopathologic investigations and appeared to be superior to standard histology with HE staining.^12^ The fact that the pooled PPV of OSNA (79.8%) was lower than other values as shown in Table 2, shows that the false‐positive rate of OSNA is high. This may be because the OSNA assay can detect some LN metastases of CRC that cannot be detected by histopathological examinations due to tissue allocation biases or insufficient performance for the detection of micrometastasis.

The studies listed in Table 2 evaluated the diagnostic accuracy, comparing the OSNA and histopathological examination in resected LNs one by one. However, given the high prevalence of CRC and the high number of LNs to be analyzed, systematic molecular LN analyses and additional diagnostic methods beyond routine HE are unlikely to be incorporated into the pathological diagnosis, largely because of the high cost of molecular techniques and the supplementary workload. To resolve this issue, Rakislova et al^16^ conducted a study comparing two methods of the OSNA for detection of LN metastasis in CRC: an individual analysis of each LN and a new approach involving pooling several LNs, known as the “pooling method.” The diagnostic performance of the OSNA with the pooling method was comparable to that of the individual analysis, and the authors concluded that the pooling strategy was a rapid method that can be routinely performed and would aid in the therapeutic management of CRC patients.

## 5 THE OSNA ASSAY FOR THE DETECTION OF CRC METASTASIS IN SLNS

In CRC, the SLN concept was introduced in 1999 by Joosten et al^27^ in order to reduce the false negative result rates and to understand the importance of the LN involvement for further therapy and the survival rate of these patients. SLNs are considered to be the LNs located closest to the tumor in the lymphatic drainage pathways, bearing the highest risk of tumor involvement.^27^ Using lymphatic mapping, two to four such LNs may be identified.

The principle of an SLN biopsy is well established in melanoma and breast cancer, where the aim is to avoid unnecessary and potentially morbid lymphadenectomy. Unlike these two malignancies, where lymphadenectomy is a separate procedure, lymphadenectomy in elective CRC surgery is typically performed as part of a single surgical procedure. Therefore, an SLN biopsy to omit unnecessary lymphadenectomy does not seem clinically useful in CRC surgery. However, if an SLN biopsy can readily and reliably determine the LN status, permitting more conservative surgery, then reduced tissue dissection, shortened operative time, and a better bowel function are all desirable outcomes, especially in early‐stage CRC patients.

The main advantage of SLN mapping in CRC appears to be the identification of nodes that carry an increased risk of metastasis, as these LNs can then be subjected to detailed scrutiny, including more sections and IHC, thereby optimizing the staging accuracy. While whether or not LN mapping can improve the outcomes for CRC patients is unclear because the influence of the detected micrometastasis on the survival and therapeutic decision is controversial, different studies have shown that ex vivo SLN mapping is an easy and feasible technique for CRC patients. This technique was characterized by a high accuracy of 90%‐100% and NPV of 80%‐100%,^28^, ^29^ and upstaging has been observed in 19%‐57% of cases.^29^ With the advent of the OSNA technique, which enables the whole‐tissue assessment of LN metastasis rapidly, the merits of the SLN concept may be reconsidered.

Previous studies analyzing CRC metastases in SLNs with the OSNA are listed in Table 3. The injected dyes for the detection of SLNs were blue dye or indocyanine green. An intraoperative OSNA assay was performed in two studies, and a postoperative OSNA assay using SLNs stored at −80°C was performed in the remaining two studies. A previous SLN analysis in colon cancer patients performed by Vogelaar et al^14^ compared the performance of an OSNA with a routine pathological examination (single HE staining) and multilevel fine pathological examination (IHC with pan‐cytokeratin antibody staining). They showed that the performance of the OSNA and fine pathological examination were superior to that of the routine pathological examination. Furthermore, an upstaging rate of 46.5% was obtained by combining the two methods together.

**Table 3:** Studies analyzing CRC metastasis in SLNs with OSNA

Author	Patients number (Sample number)	Injected dye	Intraoperative OSNA assay	The number of examined SLNs
Vogelaar et al^14^	128 (325)	Patent Blue Dye V or Indocyanine Green	No	3.0 (median)
Marhic et al^18^	17	Blue dye	Yes	Not shown
Yeung et al^21^	16 (78)	Indocyanine Green	No	4.9 (mean)
Esposito et al^23^	34 (51)	Indocyanine Green	Yes	1.0 (median)

Abbreviation: SLN, sentinel lymph node.

The average total time needed to complete the OSNA assay is approximately 40 minutes,^30^ and this length of time is acceptable for the intraoperative examination of SLNs. Two studies performed intraoperative OSNA assays to evaluate not only its diagnostic performance but also the time interval between surgery and postoperative adjuvant chemotherapy.^18^, ^23^ Although both studies were small pilot studies, they showed that an intraoperative OSNA assay was able to significantly reduce the time between surgery and adjuvant chemotherapy in node‐positive CRC patients. Recent population studies and meta‐analysis confirmed that starting adjuvant chemotherapy beyond 8 weeks postoperatively was associated with a decreased overall survival compared to initiation within 8 weeks of surgery.^31^ Although the ideal start timing of adjuvant chemotherapy could never be tested in prospective randomized controlled trials for obvious ethical reasons, in routine practice it should probably be introduced as early as possible after validation by a multidisciplinary team and according to patient recovery. In this regard, an OSNA assay, which can establish the LN status as soon as possible and minimize the risk of delayed adjuvant chemotherapy, seems to be useful in the management of high‐risk stage II and stage III CRC patients.

## 6 AN OSNA ASSAY FOCUSING ON UPSTAGING IN PATHOLOGICALLY NODE‐NEGATIVE CRC PATIENTS

Patients with stage II CRC (T3‐4N0M0) form a heterogeneous group with very different prognoses, and some patients with stage II CRC may have a worse prognosis than patients with low‐risk stage III CRC (T1‐3N1).^32^ The adjuvant treatment of patients with high‐risk stage II colon cancer (poorly differentiated histology, presence of lymphovascular invasion, presence of perineural invasion, report of <12 LNs, bowel obstruction, and localized perforation) is an area of controversy in medical oncology.^33^ Therefore, increasing attention is now being focused on identifying unfavorable prognostic biomarkers that can be used to select patients for adjuvant chemotherapy. For example, circulating tumor DNA (ctDNA) as a novel biomarker for the identification of high‐risk stage II CRC is now clinically used in Western countries, and several prospective clinical studies are ongoing.^34^ The analysis of ctDNA offers a non‐invasive method of repeatedly evaluating the genomic profile of CRC patients using blood samples. The postoperative detection rate of ctDNA ranges from 10% to 15% among patients with stage II CRC and is associated with a poor prognosis.^34^

Although the prognostic value of an OSNA analysis in stage II CRC is still unclear compared with that of ctDNA, more recent studies have hypothesized that positive results on an OSNA assay in stage II CRC patients correlate with a poor disease prognosis. Brito et al examined 753 LNs from pathologically node‐negative colon cancer patients (n = 59) by an OSNA assay and showed that 28.8% of the patients had OSNA‐positive LNs.^22^ In addition, most of the OSNA‐positive patients (88.2%) were found to have LNs with micrometastases by a CK19 copy number analysis in the OSNA assay. Although the prognosis of colon cancer patients with pathologically negative OSNA‐positive LNs was not evaluated in that study, such patients may have benefited from postoperative systemic therapy, as with pathologically node‐positive patients.

Another study by Itabashi et al evaluated the prognosis of CRC patients with pathologically negative (pStage II) OSNA‐positive LNs.^25^ A total of 15.7% (11/70) of pStage II patients had OSNA‐positive nodes, and the 3‐year disease‐free survival (DFS) of such patients was extremely poor. Taken together, these studies suggest that OSNA can detect occult metastasis, such as macrometastasis, micrometastasis, and ITCs, regardless of tissue allocation bias for pathologically node‐negative CRC patients. In addition, the detection of occult metastasis by an OSNA assay might be associated with disease recurrence and poor survival, which is consistent with the findings of previous reports analyzing other types of molecular detection not only in a meta‐analysis of retrospective studies^5^ but also in a prospective multicenter trial.^35^ The prognostic value of an OSNA in pStage II CRC patients should be confirmed in larger studies for its clinical application. In Japan, a large prospective multicenter observation study (UMIN000037532) detecting cancer cells by OSNA in node‐negative stage II CRC evaluating the 3‐year recurrence‐free survival as the primary endpoint is ongoing. The result will help optimize treatment strategies in OSNA‐positive pStage II CRC patients.

## 7 THE OSNA FOR THE PREDICTION OF LPLN METASTASIS IN RECTAL CANCER

In Western countries, preoperative chemoradiotherapy (CRT) with total mesorectal excision (TME) is the standard therapy for rectal cancer.^36^ However, in Japan, TME plus lateral pelvic LN dissection (LLND) is performed for advanced low rectal cancer.^37^ Although the advantages of LLND in terms of local control have been established, it can cause sexual disorders and urinary dysfunction due to surgery‐related damage to the pelvic plexus and the lateral ligaments of the rectum containing the neurovascular bundles.^38^ Therefore, there is an urgent need to develop ways of identifying patients, before or during surgery, who potentially have LPLN metastasis. Based on the fact that the presence of perirectal LN metastasis is one of the most significant predictive factors for LPLN metastasis,^39^, ^40^ Miyake et al attempted to perform an intraoperative OSNA assay to detect perirectal LN metastasis in order to predict LPLN metastasis in rectal cancer patients undergoing surgical resection accompanied by LLND.^17^ In their study, LPLN metastases were present in 16% of patients (4/25), and all of these patients were positive on an OSNA for perirectal LN metastasis. The OSNA assay had a sensitivity of 100%, specificity of 86%, PPV of 57%, and NPV of 100% for predicting LPLN metastasis, and the authors concluded that the OSNA of perirectal LNs might be useful for selecting candidates for omission of LLND in rectal cancer surgery.

## 8 FUTURE PERSPECTIVES

The LN status remains the most significant prognostic factor and an important component of the decision‐making process regarding the adjuvant treatment for CRC cancer patients. In that respect, OSNA can be useful as an accurate, reproducible and automated assay for standardizing the LN metastasis evaluation. In addition, the OSNA may have the potential to detect occult metastasis that cannot be detected by a histopathological examination. Furthermore, Diaz‐Mercedes et al analyzed the budget impact of introducing an OSNA assay in early‐stage CRC patients and suggested that OSNA might have not only an economic benefit but also a clinical benefit in CRC patients, since it enabled more accurate staging, thereby avoiding unnecessary treatment.^24^

However, one disadvantage of the OSNA assay is that harvesting LNs requires crude manual dissection of the fresh specimen by a trained pathologist or technician. This step is crucial for any analysis but is not uniform, so missing nodes is unavoidable. Another disadvantage of OSNA assay is that using the whole LN means there is no tissue left for a subsequent examination following complete homogenization, making it impossible to conduct multiple tests of the LN for different biomarkers in the future.

The OSNA assay is useful for determining the treatment strategy for CRC patients, as summarized above. In addition, there are several potential advantages worth evaluating in the future. For example, conducting an OSNA assay to detect LN metastasis in rectal cancer patients who have undergone preoperative CRT and surgery might be useful. The presence of residual LN metastasis despite the involutional effect of CRT has been shown to be a major risk factor of local recurrence, distant recurrence, and poor survival.^41^, ^42^ However, LNs after neoadjuvant therapy frequently show severe histological changes, including stromal fibrosis and elastosis with small clusters of residual and viable metastatic tumor cells. OSNA measures CK‐19 derived from viable tumor cells and may thus be able to assess these changes more accurately than a pathological examination. Indeed, there have been a few reports describing the utility of an OSNA assay in breast and rectal cancer patients with neoadjuvant chemotherapy.^17^, ^43^, ^44^ In addition, an OSNA assay for the detection of extended LN metastasis, such as LPLN metastasis or para‐aortic LN (PALN) metastasis, from CRC might be useful. PALN or LPLN metastasis from CRC has been shown to be a significant prognostic factor.^45^, ^46^ An accurate diagnostic tool, such as the OSNA, seems effective for determining the treatment strategy after the evaluation of extended LN metastasis, as an accurate diagnosis can be achieved rapidly (intraoperatively if necessary), regardless of the preoperative treatment.

The main advantage of an OSNA assay over a pathological examination is that it provides objective and semi‐quantitative data on the tumor volume in the whole LN rapidly with little to no effort by pathologists and with low inter‐observer variability. As described above, an OSNA assay can help improve the treatment outcome of CRC patients in several respects, such as in the detection of high‐risk stage II disease, the SLN analysis and the prediction of LPLN metastasis. Multicenter prospective studies with a large cohort of CRC patients are warranted to confirm such advantages of an OSNA assay, and technical refinements of the OSNA system are essential before it can be applied as a new standard.

## DISCLOSURES

Conflicts of interest: Authors declare no conflicts of interest or financial ties to disclosure.

Author Contribution: Yukiharu Hiyoshi study conception design, Drafting the article. Takashi Akiyoshi study conception design. Yosuke Fukunaga study conception design.
